# Genome-Wide Association Study towards Genomic Predictive Power for High Production and Quality of Milk in American Alpine Goats

**DOI:** 10.1155/2020/6035694

**Published:** 2020-07-26

**Authors:** Y. Tilahun, T. A. Gipson, T. Alexander, M. L. McCallum, P. R. Hoyt

**Affiliations:** ^1^American Institute for Goats Research, Langston University, Langston, Oklahoma, USA; ^2^Biochemistry and Molecular Biology, Oklahoma State University, Stillwater, Oklahoma, USA

## Abstract

This paper reports an exploratory study based on quantitative genomic analysis in dairy traits of American Alpine goats. The dairy traits are quality-determining components in goat milk, cheese, ice cream, etc. Alpine goat phenotypes for quality components have been routinely recorded for many years and deposited in the Council on Dairy Cattle Breeding (CDCB) repository. The data collected were used to conduct an exploratory genome-wide association study (GWAS) from 72 female Alpine goats originating from locations throughout the U.S. Genotypes were identified with the *Illumina Goat 50K single-nucleotide polymorphisms (SNP) BeadChip*. The analysis used a polygenic model where the dropping criterion was a call rate ≥ 0.95. The initial dataset was composed of ~60,000 rows of SNPs and 21 columns of phenotypic traits and composed of 53,384 scaffolds containing other informative data points used for genomic predictive power. Phenotypic association with the *50K BeadChip* revealed 26,074 reads of candidate genes. These candidate genes segregated as separate novel SNPs and were identified as statistically significant regions for genome and chromosome level trait associations. Candidate genes associated differently for each of the following phenotypic traits: test day milk yield (13,469 candidate genes), test day protein yield (25,690 candidate genes), test day fat yield (25,690 candidate genes), percentage protein (25,690 candidate genes), percentage fat (25,690 candidate genes), and percentage lactose content (25,690 candidate genes). The outcome of this study supports elucidation of novel genes that are important for livestock species in association to key phenotypic traits. Validation towards the development of marker-based selection that provides precision breeding methods will thereby increase the breeding value.

## 1. Introduction

The use of goats to convert poorly digestible fiber into high-quality meat and milk has been a mainstay since 9,000 YBP [1]. Goats provide milk to humans more than any other dairy animal [2]. Specialization in milk production has made conversion of fiber to useable human nutrients very profitable [1]. Goat milk is generally consumed at a much larger margin than cow milk worldwide. Dairy goats are comprised of 380,000 as of January 2018 [3].

Global demand for milk consumption per capita is increasing due to both population growth and consumer preferences [4]. Genome-wide association analysis exists for other milk-producing ruminants [4]. Complex genetic and metabolic networks are responsible for regulating lactation physiology [5–7] that differ between goat milk and cow milk according to the Nutritional Composition of Goat Milk Products in the U.S. [8] based on its fat, lactose, and fatty acid composition. This suggests novel differences exist in the regulatory networks in *caprines* [7].

The secretory mechanism of lactation [7, 9] is well described, and current models exist for the regulation of fat, protein, and lactose synthesis in goat mammary tissue. The chromosomal loci and the resultant expression of microRNA by the lactating mammary glands have been proposed [10]. These discoveries using high throughput genotyping and sequencing tools generate large quantities of data with moderate time and money [10].

GWAS have benefitted from genotyping technologies like that of the high-density *Goat 50K SNP BeadChip* [10]. However, identification of causative mutations related to American goat milk yield and other American goat milk traits is lacking [4]. Whole genome level sequence variants used in association analysis provide a clearer understanding for biological mechanisms associated with each quantitative trait loci (QTL) [4]. To improve genomic selection, we employed a combination with current bioinformatics software, such as *PLINK* [11] in combination with R [12], *Qlick* [13], and analysis using *SNP & Variation Suite v8.8.1* [14].

Current genotyping arrays can capture small fractions of low frequency variants for individual goats. New or custom genotyping array developments provide avenues to utilize high priority variants with GWAS [15]. Custom chips include both common variants selected to replicate the original GWAS signals and a selection of implicated regions for relevant traits by GWAS [15]. Although second array-based technologies are limited in the range of loci they can target, cost-effective, fine-mapped, low frequency alleles may support the construction of chips that foster improved statistical power detecting variants [15].

The *GoatSNP50 BeadChip* was used to determine genetic diversity of Boer, Cashmere, and Rangeland goats [16]. The success of more recent genomic studies in the dairy goat industry suggests that successful implementation of low-panel SNP assays could revolutionize goat breeding and production in the United States, as it did with dairy cattle [17].

High-density SNP data has effectively identified regions of the genome that are important predictors of production traits (i.e., [18] and [19]). The development of robust prediction equations for genomic selection often requires the use of large numbers of animals with high-quality genotypes and phenotypes. However, when numbers of samples are limited, improvements in statistical limitations can result in preferential selection of the most informative samples [15]. Quantitative traits can use much smaller sample sizes than can do random sampling [15], and the extreme costs associated with genome scale association studies sometimes require that qualitative similar data analysis uses alternative methods when including smaller sample sizes [20].

This study explored the use of genome-wide associations to identify heritable milking traits in the American Alpine goat. We hypothesize that associations would exist and could be elucidated for future development of a low-panel *SNP BeadChip* for use in research and animal management that would not be cost prohibitive for researchers and smaller scale producers.

The specific aims of the study were to

1. improve contributions to the genotype-phenotype repository of the Council on Dairy Cattle Breeding (CDCB) for milk quality traits that are economically important for goat production
2. develop genomic prediction and provide data for better tools, for predicting phenotypic traits, and for the selection of specific SNPs associated with select signatures (genes) that determine specific phenotypic traits in American Alpine goats by the initial use of the high-density *Goat50KSNP BeadChip*
3. establish whether a low number of goat subjects (<300 goats) will provide statistically significant (*p* < 0.05) predictive capabilities for desired breeding traits in American Alpine dairy goats

## 2. Materials and Methods

### 2.1. Ethics Statement

No animal experiments were performed in this study and was approved by the ethics committee (Approval # 15-117). Milk samples were collected from local, state, and national farms where goat milk samples were obtained. Sire ID, breed, somatic cell count and milk weight by test days, lactations, history of milk yield, protein percentage, and fat percentage are located in the CDCB repository.

### 2.2. Phenotypes

Phenotypic records for American Alpine goats (*Capra hircus*) were obtained from national breeding programs across the United States, including those at the Langston University-American Institute for Goat Research (LU-AIGR). Breeding values for fat, protein, lactose, solids, solids-not-fats (SNF), urea, freezing-point-depression (FPD), somatic cell count (SCC), test day milk yield (TDMilk), test day fat (TDFat), test day protein (TDProtein), test day somatic cell counts (TDSCCS), and Call_Rate were recorded (see Supplementary section (see available here)). The production data was collected from milk records contributed and indexed for each trait into the CDCB repository.

Does from throughout the United States were utilized in this study with varying phenotypes and environmental stimuli. Neither handling and feeding conditions throughout the experiment nor birth month nor season factors were included in the statistical analysis. The statistical study was carried out following two generally applied mathematical models, a mixed model and a fixed model. The mixed model is described in

(1) $$\begin{array}{c} Y_{ijklm}=\mu +L_{i}+W_{j}+A_{k}+P_{l\left(k\right)}+N_{m}+e_{jklm} \end{array}$$

where *Y*_*ijklm*_ is the test day measurement (milk, log SCC, fat percentage, protein percentage, casein percentage, serum protein percentage, lactose percentage, and total solid percentage) for a particular lactation *L*_*i*_ of a doe of birthing age *A*_*k*_ at parity *P*_*l*_ within *A*_*k*_, during postpartum week *W*_*j*_, and with *m* number goats weaned (*N*_*m*_) [21]. Lactation was the only random effect, with 155 levels. The week, age, parity within age, and number of lambs weaned were fixed effects. This mixed model was based on that applied by Stanton et al. [22] for estimating lactation curves of dairy cows for milk, fat, and protein. This model presents greater validity when used to study lactation curves with a high number of test day for lactation and could be considered a good approximation to more complex mathematical models. The lactation random effect explains more variation in test day yield than does any other factor.

### 2.3. Population Resources and DNA Isolation

Milk producers delivered milk samples in 50 ml plastic vials containing *bronopol* preservation tablets that preserved DNA in milk at ambient temperature [23]. Milk sample collections were made during the fourth peak lactation from each goat. Somatic cell counts were conducted, and the milk was subsequently stored at 4°C until DNA isolation. High-quality DNA samples were prepared within a laboratory setting at LU-AIGR utilizing Norgen's Milk DNA Preservation and Isolation Kit (NORGEN BIOTEK CORP., Thorold, ON, Canada). The quantity (ng/*μ*l) and the quality (A260/A280) of DNA samples were assessed by spectrophotometric techniques on a 96-well automated spectrophotometer (BMG Labtech, Cary, NC, USA). Genotyping assays utilizing the *GoatSNP50 BeadChip* were completed at 30x depth through the Core Facility at the Oklahoma Medical Research Foundation (OMRF). High-quality genotype assays were obtained following QC/QA determinations from 276 DNA samples derived from somatic cells contained in goat milk for use in this study. The final number of samples was reduced for SNP analyses to 72 different goats.

### 2.4. SNP Array Genotyping and GWAS

The *GoatSNP50 BeadChip* was used to identify the strongest associations of SNPs to phenotypic data. The platform used is GPL28152 Illumina Goat IGGC_conf_60K Genotyping BeadChip (GoatIGGC_conf_60K). Isolated DNA samples were hybridized on individual high-density *GoatSNP50 BeadChips*. The original corresponding goat milk samples with defined phenotypic traits were used with the reference *GoatSNP50 BeadChip* to conduct a GWAS using *PLINK* [11] to analyze our data. Minimum call rates of 95% for individuals and 95% for loci were used to select relevant SNPs. Marker loci with minor allele frequencies below the default and deviation from Hardy-Weinberg proportions were excluded. The minimal acceptable Guanine-Cytosine (GC) score was the default (0.70) for individual genotyping, and average GC scores below the default (0.75) were excluded. After quality control, this generated 42,670 SNP genotype variants out of 53,384 with 10,714 unknown variants. Different scale parameters were tested, and a scale = 4 was chosen for its best fit on data generated using R [12] and with *SNP & Variation Suite v8.8.1* [14].

### 2.5. Validation of Test Statistics with QQ Plots

**Figure 1:**
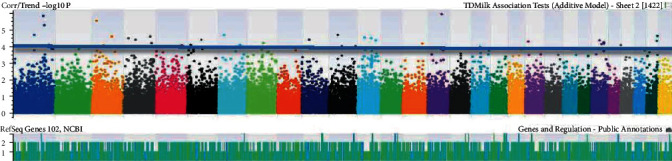
Identification of genomic coordinates on Manhattan plots associated with test day milk in the chromosomes of *Capra hircus*.

**Figure 2:**
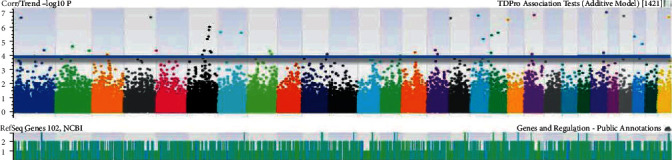
Identification of genomic coordinates on Manhattan plots associated with test day protein in the chromosomes of *Capra hircus*.

**Figure 3:**
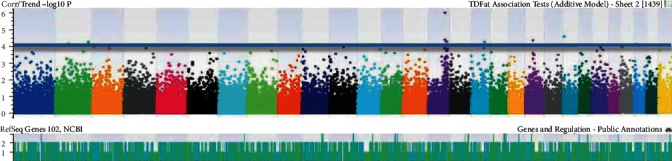
Identification of genomic coordinates on Manhattan plots associated with test day fat in the chromosomes of *Capra hircus*.

**Figure 4:**
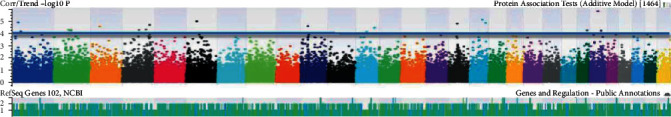
Identification of genomic coordinates on Manhattan plots associated with protein in the chromosomes of *Capra hircus*.

**Figure 5:**
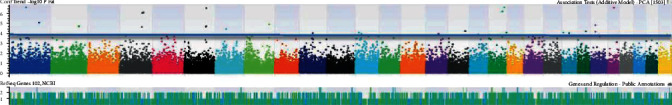
Identification of genomic coordinates on Manhattan plots associated with fat in the chromosomes of *Capra hircus*.

**Figure 6:**
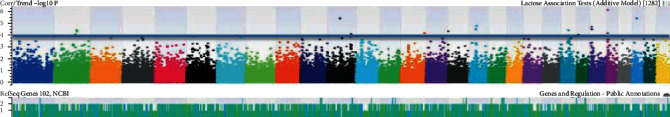
Identification of genomic coordinates on Manhattan plots associated with lactose in the chromosomes of *Capra hircus*.

The number of SNPs was identified after basic association tests correlating specific phenotypic traits of each goat. The traits were selected according to the strongest associations having *p* < 0.05. The identification of genomic coordinates on Manhattan plots (Figures 1–6) with genome-wide significance levels greater than -log_10_(*p*) = 4.0 is shown in the figures. Association with milk yield (TDMilk), protein yield (TDProtein), fat yield (TDFat), protein, fat, and lactose composition was completed, respectively. Phenotypic/genotypic associations were observed for variance, and novel SNPs with strong association for particular traits were identified that segregated for inclusion on a low-density SNP Chip.

After these basic association tests correlating specific phenotypic traits, the number of SNPs for each goat was selected based on the strongest associations having *p* < 0.05, where their negative logarithms would be the greatest. Analysis was conducted using *SNP & Variation Suite v8.8.1* [14]. Spreadsheets were generated that identified relevant associations between different data sets and were used to produce figures and charts for additional visualization of the strongest associations having *p* < 0.05.

## 3. Results

### 3.1. Frequency of Milk Phenotypes in Alpine Goats

**Figure 7:**
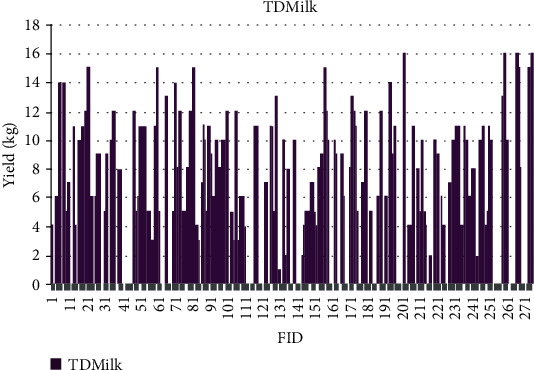
The yield values for the phenotypic trait test day milk obtained from goats in relation to their FID number (1–276).

**Figure 8:**
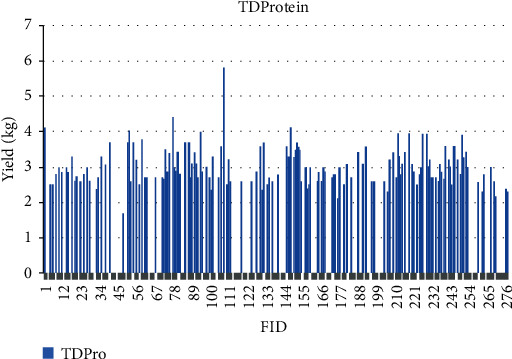
The yield values for the phenotypic trait test day protein obtained from goats in relation to their FID number (1–276).

**Figure 9:**
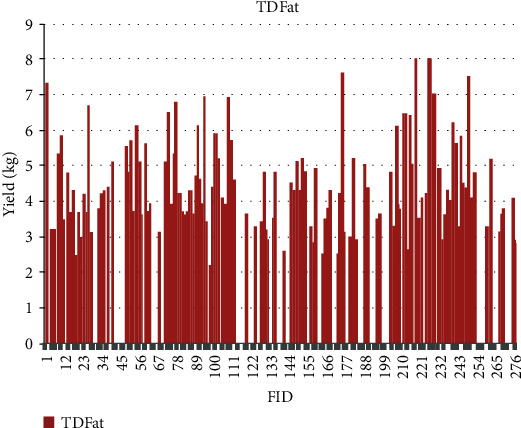
The yield values for the phenotypic trait test day fat obtained from goats in relation to their FID number (1–276).

**Figure 10:**
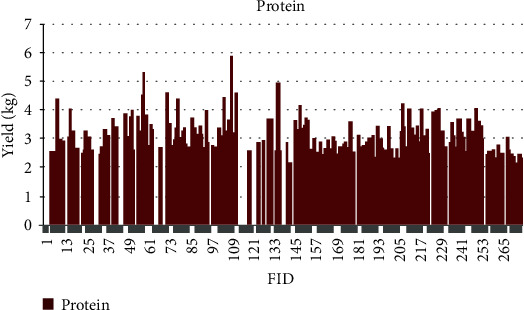
The yield values for the phenotypic trait protein obtained from goats in relation to their number FID (1–276).

**Figure 11:**
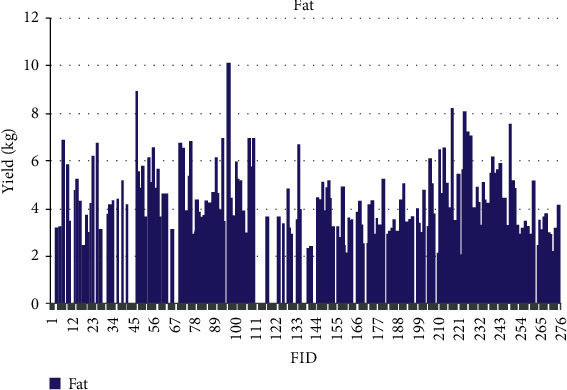
The yield values for the phenotypic trait fat obtained from goats in relation to their FID number (1–276).

**Figure 12:**
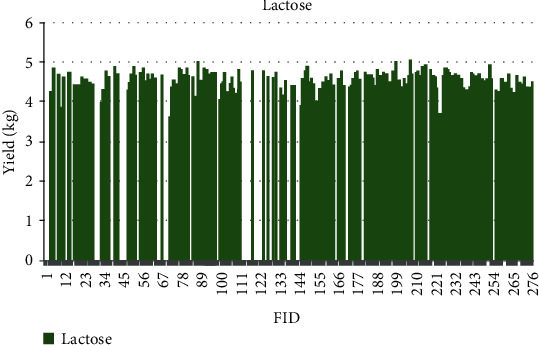
The yield values for the phenotypic trait lactose obtained from goats in relation to their FID number (1–276).

The quantitative values for phenotypic traits are depicted for each sample based on feature identification (FID) numbers in Figures 7–12. The yield values for phenotypic traits (TDMilk, TDProtein, TDFat, protein, fat, and lactose) are obtained from goats in relation to their FID (1–276).

**Figure 13:**
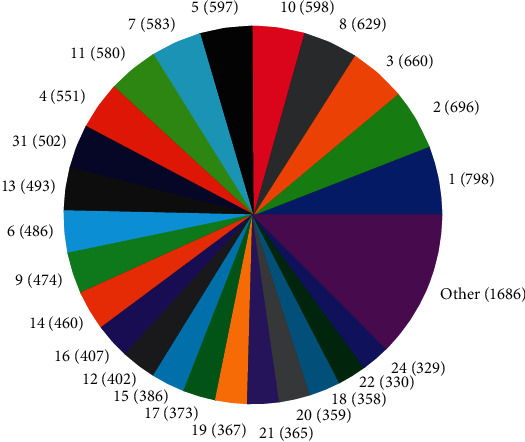
Pie chart of the number of SNPs (in parentheses) associated with test day milk yield in the chromosomes of *Capra hircus*.

**Figure 14:**
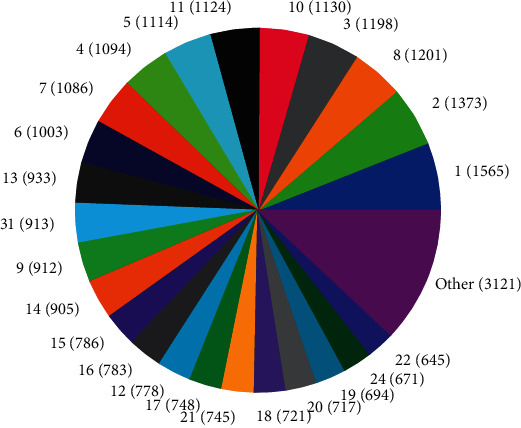
Pie chart of the number of SNPs (in parentheses) associated with test day protein yield in the chromosomes of *Capra hircus*.

**Figure 15:**
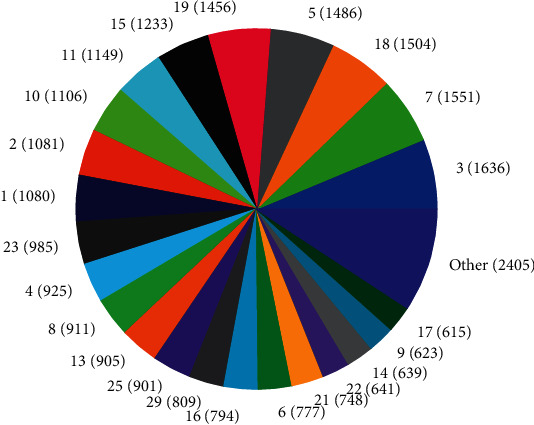
Pie chart of the number of SNPs (in parentheses) associated with test day fat yield in the chromosomes of *Capra hircus*.

**Figure 16:**
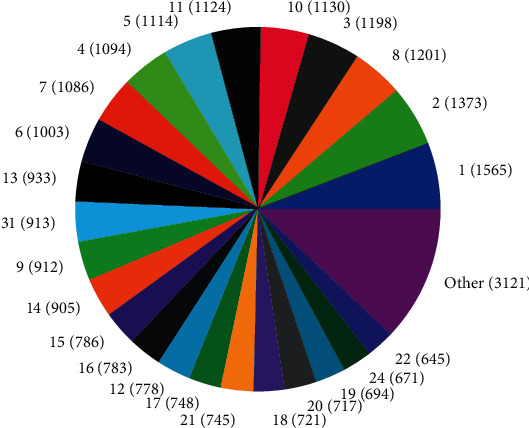
Pie chart of the number of SNPs (in parentheses) associated with protein content in the chromosomes of *Capra hircus*.

**Figure 17:**
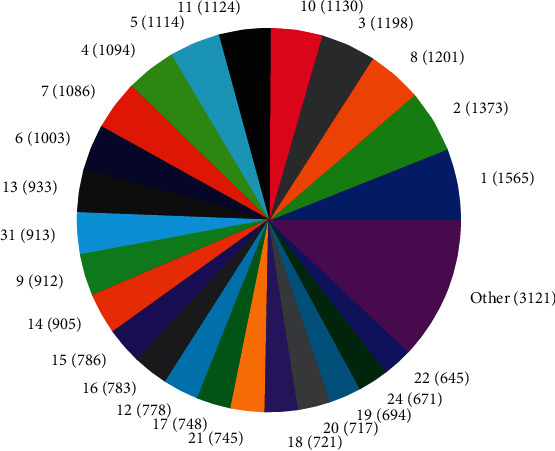
Pie chart of the number of SNPs (in parentheses) associated with fat content in the chromosomes of *Capra hircus*.

**Figure 18:**
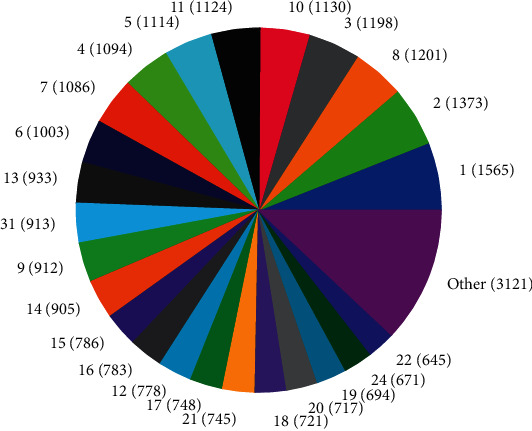
Pie chart of the number of SNPs (in parentheses) associated with lactose content in the chromosomes of *Capra hircus*.

The distributions of SNPs and the yield values for phenotypic traits (TDMilk, TDProtein, TDFat, protein, fat, and lactose) with the *50KGoatSNP BeadChip* in relation to the phenotypic traits in relation to their FID number (1–276) are shown as the numbers of SNPs (Figures 13–18) in each of the 30 chromosomes in the *Capra hircus* genome. These are shown in pie charts (note: female goat X chromosome = chromosome number 31).

The milk phenotypes and their associated genes TDMilk (*n* = 13,469), TDProtein (*n* = 25,960), TDFat (*n* = 25,960), protein (*n* = 25,960), fat (*n* = 25,960), and lactose (*n* = 25,960) in the different data sets are presented below.

The GWAS data sets of phenotypes associated with genotypic components of the *50KSNP BeadChip* are shown (Table 1). The number of statistically significant (*p* ≤ 0.05) SNPs at a call rate ≥ 0.95 was selected for approximate numbers of genotype/phenotype association (Table 1).

Quality genotypic data points were obtained after thorough quality control and quality assurance (QC/QA) [24]. This GWAS study used several advantageous procedures involving approaches that distinguish chromosome aberrations that may affect genotype accuracy and detect genotyping artifacts from dependence of Hardy-Weinberg equilibrium (HWE) [24, 25] test *p* values. Allelic frequency of the *p* values as well as the capability of selecting SNPs, from demonstrated principal component analysis using HWE, showed that most SNPs are associated with the traits in this GWAS.

The identification of genomic coordinates on Manhattan plots (Figures 1-6) with genome-wide significance levels (−log_10_(*p*) = 4.0) was completed for test day milk, test day protein, test day fat, protein, fat, and lactose, respectively. Phenotypic/genotypic associations were identified for variance, and novel SNPs with strong association for particular traits were segregated for inclusion on a low-density SNP chip. Manhattan plots for these six analyses indicated several significant regions at the genome threshold indicating outliers and chromosome level, niche association with specific, phenotypic traits.

**Figure 19:**
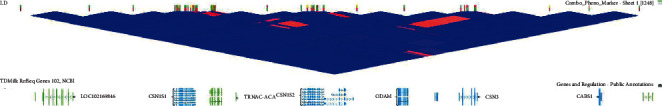
Identification of genomic coordinates (Combo_Pheno_Marker) using a portion of a heat map associated with TDMilk in comparison to RefSeq genes in National Center for Biotechnology Information (NCBI). The chromosomes of *Capra hircus* were used to for association with known sequences of SNPs. LOC102169846 is associated with estrogen sulfotransferase. CSN1S1 is associated with casein alpha s1. ODAM is an ameloblast associated with a processed peptide: odontogenic ameloblast-associated protein. CABS1 is associated with a calcium-binding protein. These and many more associated SNPs identified by this study are found in the NCBI repository.

Figure 19 serves as an example of the many areas of identification of genomic coordinates specifically compared to known genes in the National Center for Biotechnology Information (NCBI) repository.

## 4. Discussion

### 4.1. Purpose

Custom-designed genotyping tools that are smaller and are less costly than the original, high-density tools have similar predesigned amounts of computational power. The selection criteria include, but are not limited to, small *p* values (^∗^*p* < 0.05), large estimated effect sizes (odds ratios), and previous reports of association. They also include biologically relevant pathways, plausible function of variations, or a combination of all these factors.

This is an exploratory study and did not analyze high probability rare variants. Previous studies show that 460 to 4,600 individuals are needed to assume certainty for detections [15]. Also, Lee et al. [15] mentioned that the detection of rare-variant associations is underpowered when involving standard single-variant association analysis. Complex traits are predicted using genomic selection (GS) where information involving thousands of genetic markers from genomic signatures is utilized [26]. This exploratory study proposes that a more specific method be used to identify salient genomic signatures.

The goals of this exploratory study were to identify regions of the Alpine goat genomes that are important for predicting genetic merit in female dairy goats. This is useful to develop genomic prediction equations for the genetic evaluation of economically important milk production traits (TDMilk, TDProtein, TDFat, protein, fat, and lactose). This would further identify methods for high-yield and high-quality milk towards the production of goat milk, cheese, ice cream, and many other dairy goat products for the consumer through the producer. The selection of goat phenotypes could be predicted while it is young, before reaching the age of gestation, thereby facilitating appropriate use of resources and positively leading to selecting genetic change in goat herds.

These approaches comprise the first step in identifying SNPs for future production of a low-density SNP panel (possibly a *5K BeadChip*) available for goat producers at a reduced cost, compared to the current cost of a *50K BeadChip*. This would facilitate both genetic selection and integration of genomic technologies into production systems, contributing to precision agriculture.

### 4.2. Discoveries

#### 4.2.1. Genomic Variability

This study identified information that can be used for the benefit of genomics in goats and other organisms. Secondary to the identification of defined SNPs associated with the traits identified, this study suggests what may be a new direction to understand how organisms within the same species utilize different pathways while effectively using the same SNPs. As the pie charts in Figures 16–18 indicate, the same number of SNPs associates to the same chromosomes. However, a closer look reveals they are of the same quantity but not of the same composition (same number of SNPs but different SNPs). Many explanations for these data sets (the results involving possible indels, substitutions, translocations, and other chromosomal, stable aberrations) suggest a more complex system than initial identification of quantity provides. Because these exist, within the same breed of female American Alpine goats, more detailed studies are warranted.

Onzima et al. [27] show that little overlap occurs between breed genomic regions of breeds that are under selection. This is because traits are complex and do not always display classic signatures of selection. Onzima et al. [27] mention that the processes of complex networks of genes acting together to environmental stressors are what lead to diverse biological, molecular, and cellular functions. They also suggest that most signatures are breed specific. Although this study was small in comparison to other GWAS, it is still remarkable that it supports the idea that breed specificity, a driving force in the genomic composition, can be explained as part of adaptation to varied environmental conditions [27].

Another explanation comes from Keller et al. [28], who described that variation can occur due to the stochastic nature of genomes caused by recombination of chromosomes in autosomal cells. Furthermore, the different frequencies that are observed within the same breed could be due to different population structures, population history, and use of selection strategies [29]. Brito et al. [30] made similar propositions for variability that in general, the breed development history impacted genes because they were potentially under selection.

### 4.3. Yield Variability

Another area of interest is the variability that appears in yield as it relates to the phenotypes under observation in this study. The variability in yield among the individual traits within the same breed of goat is as follows: lactose < protein < TDProtein < TDFat > Fat < TDMilk. Although there are several studies considering variability from a genomic aspect, there are not many that refer to the effects of the genomic composition and selection of the animals on their trait variability.

## 5. Conclusion

Although this study is exploratory and is based on low numbers of samples, it strongly suggests an association between rare variants with traits of interest. To carry out a conclusive study for rare-variant studies on Alpine dairy goats, genotyping by sequencing or genotyping much larger numbers of individuals is ideal. Nevertheless, this study shows robust associations of signals and has undertaken the discovered variants or genes to molecular or cellular functions for over 25,960 distinct SNPs that are associated with specific traits expressed by American *Capra hircus* or Alpine dairy goats.

The amount of genotypic data in this study was relatively high, considering the low number of subjects. It enabled the discovery of multiple regions of the genome that were associated at chromosome-wide significance levels with desired traits from American Alpine dairy goats. A combined approximation of 30,594 SNPs was detected for six phenotypic traits at levels of significance (^∗^*p* < 0.05), respectively. These results imply that the possibility of a marker-based selection of desired traits is possible.

Currently, there is a *50K SNP BeadChip* that is available to identify those goats that contain the genes for desirable traits examined here. The creation of a 5K or less SNP chip for the traits described in this study can provide similar information about each goat and would be a better way of precision breeding while preserving resources for better use in a goat-producing farm or facility. A larger scale study is cleared indicated to more conclusively identify DNA fragments for inclusion on a low-panel SNP chip. Finally, additional insight as to the relationship of phenotypic/genotypic effects on the variability in yields should be examined.

## Figures and Tables

**Table 1:** Number of genes from American Alpine goats in association with genotypic components of the *Goat SNP50 Goat BeadChip*.

Component	# of total value	# of distinct value	# of SNPs (p ≤0.05)
Candidate genes	60,000	26,074	
Test day milk	26,074	13,469	~1,110
Test day protein	26,074	25,960	~11,994
Test day fat	26,074	25,960	~1,324
Protein	26,074	25,960	~1,404
Fat	26,074	25,960	~1,590
Lactose	26,074	25,960	~13,172

## Data Availability

All relevant supplementary and raw data are available on the National Center for 427 Biotechnology Information–Gene Expression Omnibus (NCBI-GEO) website 428 (https://www.ncbi.nlm.nih.gov/geo/query/acc.cgi?acc=GSE145419) (BioProject 429 PRJNA607190, GEO Accession GSE145419).

## Supplementary Materials

Supplementary Materials PLINK_060317_0949 iSelect p1-5 (Yonathan goat p1-5) 2017-03-03.bat iSelect p1-5 (Yonathan goat p1-5) 2017-03-03.map iSelect p1-5 (Yonathan goat p1-5) 2017-03-03.ped iSelect p1-5 (Yonathan goat p1-5) 2017-03-03.phenotype iSelect p1-5 (Yonathan goat p1-5) 2017-03-03.script Tilahun goat 276 iselect 2017-03-03_concordance.xlsx Tilahun goat 276 iselect 2017-03-03_DNAReport.csv Tilahun goat 276 iselect 2017-03-03_FinalReport.txt Tilahun goat 276 iselect 2017-03-03_FinalReportMatrixTopStrand.txt Tilahun goat 276 iselect 2017-03-03_IDs.csv Tilahun goat 276 iselect 2017-03-03_LocusSummary.csv Tilahun goat 276 iselect 2017-03-03_LocusXDNA.csv.

## References

[1] Amills M., Jordana J., Zidi A., Manuel J. (2012). Genetic Factors that Regulate Milk Protein and Lipid Composition in Goats. *Milk Production-Advanced Genetic Traits, Cellular Mechanism, Animal Management and Health*.

[2] Haenlein G. F. W. (2004). Goat milk in human nutrition. *Small Ruminant Research*.

[3] United States Department of Agriculture (2018). *National Agricultural Statistics Service*.

[4] Iso-Touru T., Sahana G., Guldbrandtsen B., Lund M. S., Vilkki J. (2016). Genome-wide association analysis of milk yield traits in Nordic Red Cattle using imputed whole genome sequence variants. *BMC Genetics*.

[5] Rudolph M. C., Neville M. C., Anderson S. M. (2007). Lipid Synthesis In Lactation: Diet And The Fatty Acid Switch. *Journal of Mammary Gland Biology Neoplasia*.

[6] Andres A.-C., Djonov V. (2010). The mammary gland vasculature revisited. *Journal of Mammary Gland Biology and Neoplasia*.

[7] Shi H., Zhu J., Luo J. (2015). Genes regulating lipid and protein metabolism are highly expressed in mammary gland of lactating dairy goats. *Functional & Integrative Genomics*.

[8] Pennington J. A. T., Church H. N. (1985). *Bowes and Church’s Food Values of Portions Commonly Used*.

[9] Mobuchon L., Marthey S., Boussaha M., Le Guillou S., Leroux C., Le Provost F. (2015). Annotation of the goat genome using next generation sequencing of microRNA expressed by the lactating mammary gland: comparison of three approaches. *BMC Genomics*.

[10] Amills M. (2014). The Application Of Genomic Technologies To Investigate The Inheritance Of Economically Important Traits In Goats. *Advances in Biology*.

[11] Purcell S., Neale B., Todd-Brown K. (2007). PLINK: a tool set for whole-genome association and population-based linkage analyses. *American Journal of Human Genetics.*.

[12] R Core Team R A language and environment for statistical computing. R Foundation for Statistical Computing. https://www.R-project.org/.

[13] Qlik Sense [Software] *PA: Qlik Technologies Inc, King of Prussia*.

[14] *SNP & Variation Suite (Version 8.8.1) [Software]*.

[15] Lee S., Abecasis G. . R., Boehnke M., Lin X. (2014). Rare-variant association analysis: study designs and statistical tests. *The American Journal of Human Genetics*.

[16] Kijas J. W., Ortiz J. S., McCulloch R. (2013). Genetic diversity and investigation of polledness in divergent goat populations using 52 088 SNPs. *Animal Genetics*.

[17] Nayeri S., Sargolzaei M., Abo-Ismail M. K. (2016). Genome-wide association for milk production and female fertility traits in Canadian dairy Holstein cattle. *BMC Genetics*.

[18] McCue M. E., Bannasch D. L., Petersen J. L. (2012). A high density SNP array for the domestic horse and extant perissodactyla: utility for association mapping, genetic diversity, and phylogeny studies. *PLOS Genetics*.

[19] Sánchez-Molano E., Woolliams J. A., Pong-Wong R., Clements D. N., Blott S. C., Wiener P. (2014). Quantitative trait loci mapping for canine hip dysplasia and its related traits in UK labrador retrievers. *BMC Genomics*.

[20] Li Y., Levran O., Kim J. J., Zhang T., Chen X., Suo C. (2019). Extreme sampling design in genetic association mapping of quantitative trait loci using balanced and unbalanced case-control samples. *Scientific Reports*.

[21] Barillet F. Expression de la production laitiere a la traite des brebis Lacaune en systeme allaitement x traite me’canique.

[22] Stanton T. L., Jones L. R., Everett R. W., Kachman S. D. (1992). Estimating milk, fat, and protein lactation curves with a test day model. *Journal of Dairy Science*.

[23] Newton A., Williams C., Martin A. L., Tilahun Y., Zeng S. (2018). *Quantitative and qualitative improvements in DNA extraction procedures using a Bronopol TM tablet in Alpine goat milk*.

[24] Laurie C. C., Doheny K. F., Mirel D. B. (2010). Quality control and quality assurance in genotypic data for genome-wide association studies. *Genetic Epidemiology*.

[25] Haldane J. B. S. (1954). An exact test for randomness of mating. *Journal of Genetics*.

[26] Lenz P. R. N., Beaulieu J., Mansfield S. D., Clément S., Desponts M., Bousquet J. (2017). Factors affecting the accuracy of genomic selection for growth and wood quality traits in an advanced-breeding population of black spruce (Picea mariana). *BMC Genomics*.

[27] Onzima R. B., Upadhyay M. R., Doekes H. P. (2018). Genome-wide characterization of selection signatures and runs of homozygosity in Ugandan goat breeds. *Frontiers in Genetics*.

[28] Keller M. C., Visscher P. M., Goddard M. E. (2011). Quantification of inbreeding due to distant ancestors and its detection using dense single nucleotide polymorphism data. *Genetics*.

[29] Forutan M., Mahyari S. A., Baes C., Melzer N., Schenkel F. S., Sargolzaei M. (2018). Inbreeding and runs of homozygosity before and after genomic selection in North American Holstein cattle. *BMC Genomics*.

[30] Brito L. F., Kijas J. W., Ventura R. V. (2017). Genetic diversity and signatures of selection in various goat breeds revealed by genome-wide SNP markers. *BMC Genomics*.

